# Implementation of online peer feedback for student self-reflection – first steps on the development of a feedback culture at a medical faculty

**DOI:** 10.3205/zma001250

**Published:** 2019-08-15

**Authors:** Bianca Raski, Alexander Eissner, Elisabeth Gummersbach, Stefan Wilm, Linn Hempel, Melina Dederichs, Thomas Rotthoff

**Affiliations:** 1 Heinrich Heine University Düsseldorf, Faculty of Medicine, Office of the Dean of Studies, Düsseldorf, Germany; 2 Heinrich Heine University Düsseldorf, Institute of General Medicine, Düsseldorf, Germany; 3 Heinrich Heine University Düsseldorf, Düsseldorf, Germany; 4 Augsburg University, Medical Faculty, Department for Medical Education and Educational Research, Augsburg, Germany

**Keywords:** feedback, feedback culture, culture of trust, competence development, peer feedback, curriculum development, change measures

## Abstract

**Objective:** Acquisition and application of professional and personal competences is accompanied by the formation and consolidation of attitudes and values and is influenced by the norms and moods (trust and feedback culture) experienced in the learning environment in question [1]. In particular, feedback and peer feedback can have a positive influence on the learning progress and personal development of students [2], [3], [4]. The promotion of a culture of teaching and ultimately of trust or feedback, plays a special role in this [5]. The aim was therefore to structurally integrate feedback into the curriculum of a model study course in order to develop a feedback culture in which students can develop personally and professionally with the help of regular and constructive feedback.

**Methodology: **Following an initial pilot phase in 2009, (peer) feedback was gradually integrated into the curriculum at the medical faculty, in the first instance through checklists and subsequently through an online questionnaire and direct interviews. The activities were regularly analyzed on the basis of student evaluations using the EvaSys evaluation software and semi-standardized questionnaire-based interviews with six students in 2009 and 13 students each in 2012 and 2013.

**Results: **Initially, students felt that the trust and feedback culture at their location as being in need of improvement. There were uncertainties regarding the use of constructive feedback and making criticism but also trust issues regarding the expression of personal perceptions to faculty members. It was possible to document the increase in the acceptance of the offers in the course of their establishment by an improvement in student evaluation and an increase in the number of participants in the voluntary offers amongst others. Qualitative data showed that students had a more positive perception or assessment of the location’s feedback concept as well as indications of improvements in the culture of trust at the location. The proportion of constructive free-text comments increased significantly by 11% to 99.4% compared to the previous year (t(3)=-3.79, p=0.04). Thus, in terms of the objective, an increase in feedback activities and their quality at the faculty was achieved.

**Conclusion: **Feedback, its acceptance as well as the quality, can be positively influenced at a faculty. Change measures should be tested repeatedly in discussion with users regarding practicability in order to directly pick up implementation issues and obstacles so they can be remedied in the interests of the users. This can influence the development of a culture of trust and feedback and should promote the personal and professional development of students in the long term.

## Zusammenfassung

**Zielsetzung: **Das Erlernen und Anwenden von fachlichen, aber auch persönlichen Kompetenzen geht mit der Formung und Festigung von Einstellungen und Werten einher und wird durch die, in der spezifischen Lernumgebung gelebten, Normen und Stimmungen (Vertrauens- und Feedbackkultur) beeinflusst [1]. Insbesondere Feedback und auch Peer-Feedback können hier einen positiven Einfluss auf den Lernfortschritt und die persönliche Entwicklung von Studierenden haben [2], [3], [4]. Der Förderung einer Lehr- und letztlich Vertrauens- bzw. Feedbackkultur kommt dabei eine besondere Rolle zu [5]. Ziel war es deshalb, Feedback strukturiert in das Curriculum eines Modellstudiengangs zu integrieren, um darüber eine Feedbackkultur zu entwickeln, in der Studierende sich mit Hilfe von regelmäßigem und konstruktivem Feedback persönlich wie auch fachlich weiterentwickeln können.

**Methodik: **Nach einer initialen Pilotphase 2009 wurde (Peer-) Feedback zunächst anhand von Checklisten, später durch einen Online-Fragebogen und direkte Gesprächssituationen sukzessive in das Curriculum an der Medizinischen Fakultät integriert und die Aktivitäten regelmäßig auf Basis studentischer Evaluationen mittels der Evaluationssoftware EvaSys sowie halbstandardisierter, leitfragengestützter Interviews mit sechs Studierenden in 2009 und jeweils 13 Studierenden in 2012 und 2013 analysiert.

**Ergebnisse: **Studierende nahmen die Vertrauens- bzw. Feedbackkultur am Standort zunächst noch verbesserungswürdig wahr. Es zeigten sich Unsicherheiten mit Blick auf die Verwendung von konstruktivem Feedback und dem Geben von Kritik, aber auch Misstrauen mit Bezug zur Äußerung persönlicher Wahrnehmungen gegenüber Fakultätsmitarbeitern. Die Steigerung der Akzeptanz der Angebote im Verlauf ihrer Etablierung konnte u.a. über eine Verbesserung der studentischen Evaluation und eine Erhöhung der Teilnehmerzahlen an den freiwilligen Angeboten dokumentiert werden. In qualitativen Daten zeigte sich unter den Studierenden eine positivere Wahrnehmung bzw. Bewertung des standorteigenen Feedbackkonzepts sowie Hinweise auf Verbesserungen der Vertrauenskultur am Standort. Der Anteil konstruktiver Freitextkommentare stieg signifikant um 11% auf 99,4% im Vergleich zum Vorjahr (t(3)=-3,79, p=0,04). So konnte im Sinne der Zielsetzung schon eine Steigerung von Feedbackaktivitäten sowie deren Qualität an der Fakultät erreicht werden.

**Schlussfolgerung: **Feedback, dessen Akzeptanz sowie die Qualität können in einer Fakultät positiv beeinflusst werden. Dabei sollen Veränderungsmaßnahmen immer wieder im Diskurs mit den Anwendern auf Praktikabilität geprüft werden, um Umsetzungsschwierigkeiten und Hemmnisse direkt wahrzunehmen und im Sinne der Anwender zu beeinflussen. Dies kann die Entwicklung einer Vertrauens- bzw. Feedbackkultur beeinflussen und sollte langfristig die persönliche und fachliche Entwicklung Studierender fördern.

## 1. Introduction

Skills acquisition is accompanied by the formation and consolidation of attitudes and values [1]. The teaching mission statement of the Medical Faculty of the Heinrich Heine University of Düsseldorf [6], taking into account the National Competency Based Learning Objective Framework for Medicine (NKLM 2015, [http://www.nklm.de]), states that the aim is not only to promote the development of subject-related skills in students but also on a personal level [7]. Information in the form of feedback on one’s own behavior, one’s effect on others and one’s own abilities can help further develop the personal and social competencies of students, such as self-reflection, empathy, tolerance of ambiguity and the ability to cope with criticism, which are an important part of the professional identity of medical professionals [8]. Information or constructive criticism, such as feedback, is essential for the improvement of competences [9], which is why feedback as a formative examination element is an important international component in the competence-oriented framework for medical education, further education and training [10]. In this context, feedback is seen as a key element in the development of top performance [11]. The basis of this report is a definition of feedback based on Hattie and Timperley, which describes feedback as information of an intermediary (e.g. peer) regarding aspects of the performance or the comprehension of a feedback recipient regarding a specific behavior that has been exhibited [5]. However, the inclusion of direct feedback in frameworks, curricula and regulations does not necessarily mean its implementation is successful in the sense that feedback is constructively given or accepted and leads to a change in behavior. Archer points out that effective feedback within the framework of medical education can not only be achieved through absolving a prescribed number of feedback situations but must be internalized by the persons involved and lived out within a feedback culture setting [12]. An “open and constructive feedback culture” plays a key role in this and, in order to be effective, feedback must be anchored in the teaching and learning environment [11], [13]. Experts suggest that direct, personal feedback can only produce maximum benefit for students within the framework of an established feedback culture [13]. Ideally, high feedback quality is associated with both support in the use of feedback (such as training and coaching) and the need for feedback to be felt in the organization [14]. It requires a mutually appreciative common ground which in this culture realizes the giving and taking of feedback as a beneficial act for each individual, with all its various preconditions. 

According to the literature, in addition to a trust relationship between the feedback provider and the recipient [15], the learning environment (that is, the teaching/learning environment) in which the feedback is given [16] is decisive. Learning always takes place in a specific environment and is influenced by the values, moods and modes of behavior exhibited in it [1], [11]. These lived values and behaviors appear to be particularly important for formative, process-oriented examinations, which aim to constructively support the individual learning process and competence acquisition of students.

A stable feedback culture implies an attitude towards criticism, dissonance, learning and change [17] which is capable of consensus and which must be experienced by every member of the faculty before it can be further developed.

The academic community is all too familiar with issues of evaluation and feedback in higher education [18]. Meta-analyzes show that students often do not understand the requirements of feedback, do not engage with feedback and often are dissatisfied with feedback systems and processes. Moreover, there is little research on how postulated models for dealing with feedback can be put into practice [18]. Thus each location should consider the development of an improved trust culture as a lifelong process under continual change. An investigation by Schaufeli et al. suggests that a more holistic, socially embedded conceptualization of feedback and engagement is needed. This conceptualization should encourage a more productive approach to supporting students. This means providing a way to use feedback to develop learning instead of mechanistically responding to instruction [19].

**Figure 1:**
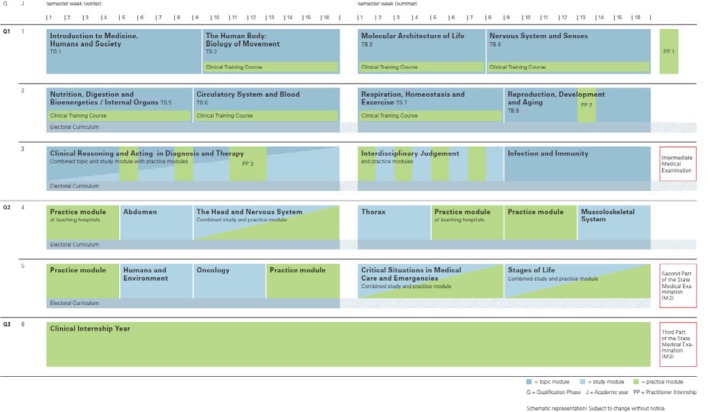
Düsseldorf Medical Curriculum (model study course)

However, feedback in the form of continual feedback which is satisfactory for students regarding their own strengths and weaknesses was still inadequate from the students’ point of view prior to the implementation of the Düsseldorf model study course in 2013 [20], [21]. The partly lacking, partly vague formulation of suggestions for improvement was also criticized [20], [21]]. In studies on the learning climate [20], [21] students as well as teaching staff assessed the existing trust culture as being in need of improvement. The aim of an initial “Feedback” project group was therefore to integrate feedback in a structured and beneficial manner into the competence-oriented medical curriculum in Düsseldorf (model study course, see figure 1 (Fig. 1)). In doing so, feedback can support teaching the following over-arching learning objectives of the Düsseldorf curriculum:

The students:

- ... contribute constructively to teams, perceive different points of view of team members and reflect on their own role (communicative competence)
- ... notice breakdowns in communication and deal with them constructively (communicative competence)
- ... notice divergences between their own values and interests and those of patients and their relatives and take them into account appropriately (communicative competence)
- ... identify and estimate their own potential with regard to the team and consider the effect of themselves on others (self-competence)

This report deals with the implementation of anonymous peer feedback which aims to

encourage students to reflect and which should support their personal and 

professional development.

The effectiveness of feedback between students (peer feedback) has been well documented in the scientific literature [12]. Since, as already mentioned, feedback culture was previously considered to be weak at the location [20], [21] a common basis characterized by esteem was considered desirable in order to be able to use self and third-party assessments implicitly and explicitly embedded with the aim of promoting student skills.

Peer feedback is used as a tool for the development of students’ higher-level competencies and interested students participated voluntarily in this project. The basic idea behind questionnaire-based peer feedback is that students are able to give each other anonymous feedback on specific behaviors or topics they observe. Anonymous peer feedback was chosen to increase the acceptance of students and to facilitate access to feedback.

## 2. Implementation and methods

### 2.1. Pilot phase

To support the development of a feedback culture at the Medical Faculty of the Heinrich Heine University in Düsseldorf, a pilot project was started in 2009, during which lecturers were asked to provide students with feedback on everyday clinical activities using checklists in combination with free text comments. 

In order to understand the obstacles faced by teachers and students in the multi-dimensional communication process of giving and receiving feedback, external educational researchers conducted a scientific follow-up evaluation with semi-standardized, guided interviews (duration 50 minutes to 2.5 hours) with open questions on implementation barriers with five physicians and six students in their Practical Year [22]. The interviews were recorded, transcribed verbatim and had their qualitative content analyzed according to Mayring [23] using the program MaxQDA version 10. Since the accompanying research suggested obstacles in the giving of direct and constructive feedback (see below) [22], initially anonymous online peer feedback was used to provide an opportunity to formulate feedback in a more secure environment without the pressure of rapid verbalization and to prevent feelings of being observed or tested. In this way feedback culture would initially be developed primarily via the target group: students. Starting in the summer semester 2012, peer to peer feedback has been implemented for students, which aimed to lower the bar for student participation. Anonymous peer feedback was chosen to allow maximally honest assessment by students and to encourage self-reflection. In direct feedback discussions, students often do not wish to burden their mutual relationships with negative assessments and in some cases do not give honest feedback [2]. Feedback givers who wish to avoid conflict that may be associated with feedback have a desire for anonymity and would otherwise tend to hold back if in doubt [24]. Acceptance of feedback by the recipient is a second important goal, so that relevant feedback does not fail due to emotional resistance by the recipient. Various studies have shown that anonymous and direct peer feedback can have a positive impact on the learning progress of students, as it is more likely to be accepted [2], [3], [4]. Anonymous feedback therefore seemed more appropriate, especially in a culture where trust was not yet stable. This procedure was chosen so that students could gain experiences with feedback which offers opportunities for improvement and is not sugar coated due to a fear of voicing criticism. The development of a culture of trust and feedback requires the promotion of learning and change abilities of an organization. Such peer feedback aims to enable students to engage in open and constructive discussion [25]. In order to establish a corresponding culture of esteem and to reduce inhibition thresholds through increased practice in giving and taking feedback, peer feedback has been successively used on a project basis in the curriculum in previous semesters. Together with the peer feedback participants as well as students who chose not to participate and following a positive vote of the ethics committee in the summer semester 2014 concrete steps for the improvement of the offer were developed in semi-standardized questionnaire-based interviews with open questions. As a result of the recorded conversations, following transcription and qualitative content analysis using the program MaxQDA version 10 [2], it was possible to establish new approaches for better information and anchoring of the project.

#### 2.2. Conducting the peer feedback

**Figure 2:**
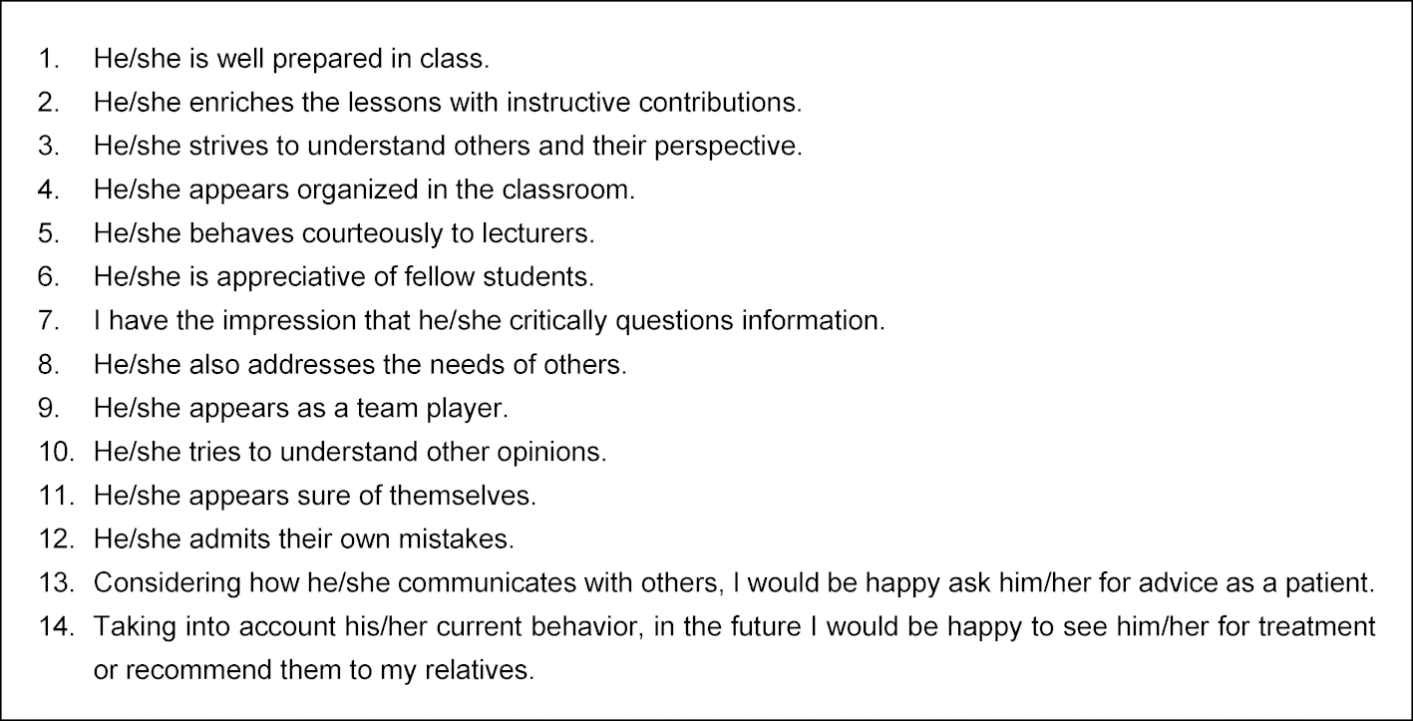
Questions about the external assessment

Peer feedback uses a specifically developed questionnaire based on the Toolkit for Lifelong Learning by Indiana University, USA [26]. This questionnaire contains items on observable peer behavior with regard to their actions in the student as well as the medical working environment (see figure 2 (Fig. 2)). Since the start of the model study course (winter semester 2013/2014), students were prepared for giving feedback with the help of curriculum-based teaching units. For example, at the start of their studies in medical psychology, students conduct simulated doctor-patient conversations with patient actors and are thereby trained in giving and receiving feedback with feedback rules and their concrete effects. Initially, feedback was conveyed using the sandwich method. Possible dilution of the actual message but also the fact that wrapping criticism with praise was often perceived as artificial by the students and met with resistance, led the project team to reject the sandwich method. By now, location-specific feedback rules were developed by a specially established working group and which represent experience-based consensus of the members in dealing with feedback and are based on the relevant literature in this area [9], [10], [5], [12]:

- Establish the framework conditions for the feedback situation.
- Explicitly name observations without evaluating them.
- Describe your subjective perception of observation.
- Illustrate potential consequences of the observed performance.
- Give suggestions for improvement related to the specific observation.
- Illustrate the benefit of your suggestions for improvement.

**Figure 3:**
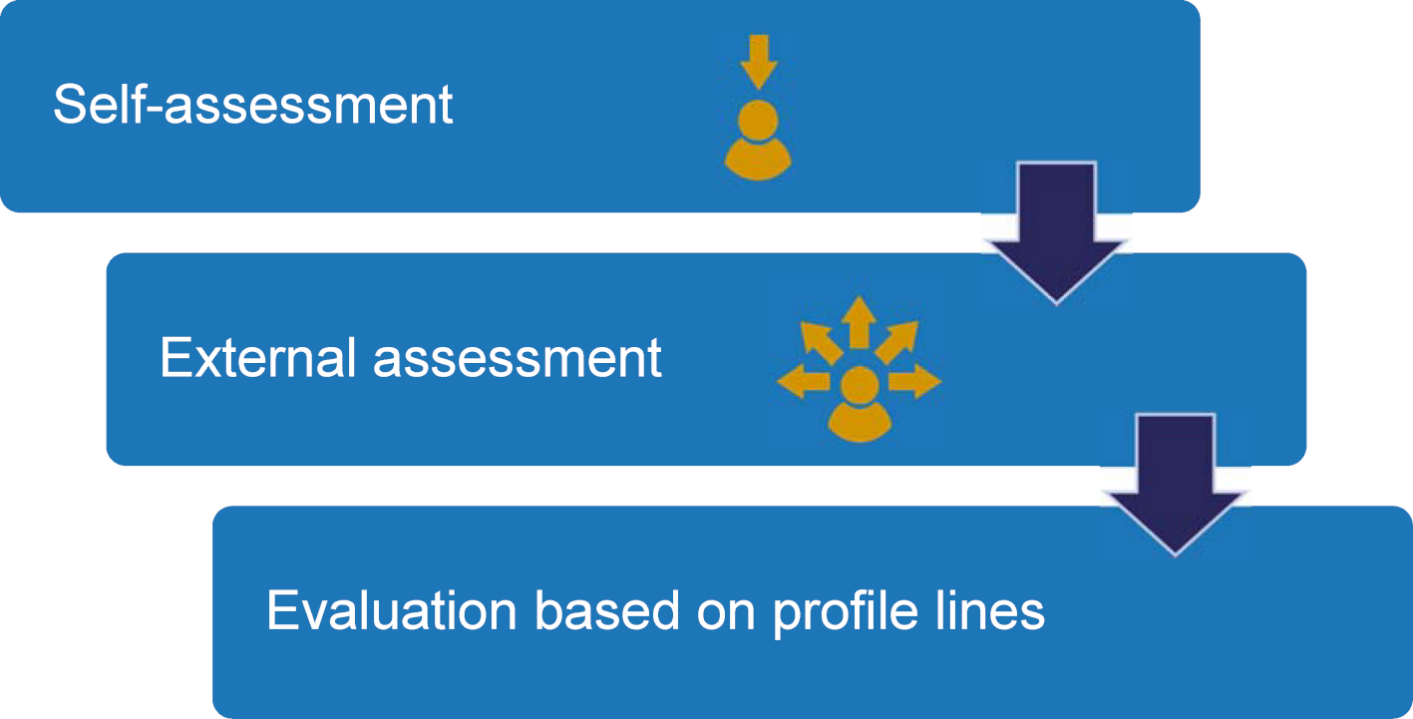
Schematic of the peer feedback process and overview of the results

**Figure 4:**
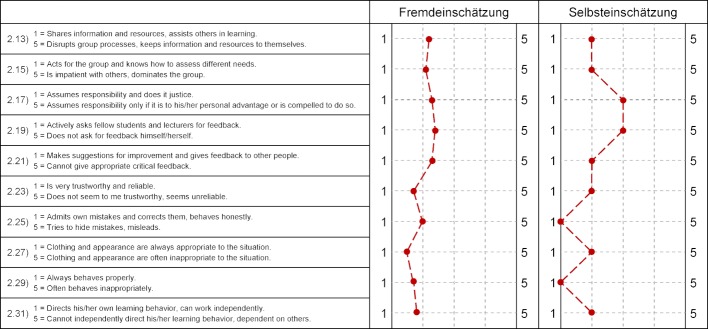
Exemplary evaluation of the peer feedback based on profile lines

This peer feedback was first offered in the summer semester 2011 on a voluntary basis and subsequently expanded. The individual assessments of each individual student are made on a five-point Likert scale (“completely agree” (5) to “do not agree at all” (1)) by the students who work together in small groups of 15 for one semester and documented using EvaSys® online evaluation software. So each student assesses their group members individually using the questionnaire and in an ideal scenario also receives such an assessment from each group member. In addition, constructive free-text comments can and should be given for each item. Two independent raters analyze the students’ free text comments on to their fellow students according to previously agreed assessment criteria (constructiveness, seriousness, discreditation). The external assessment is preceded by a self-assessment of each participant using the same items, which can then be compared to the external assessment (see figure 3 (Fig. 3)). The time required for the individual peer assessment within each group is about two to three hours each semester for each student. At the end, the students receive the cumulative and averaged assessments of their fellow students. In addition to the gender of the feedback giver and the relationship intensity as perceived by the feedback giver, the results for each item are displayed descriptively and graphically as a histogram. In addition, for direct comparison of self- and external assessments, a result profile line is drawn up for the items of both questionnaires (see figure 4 (Fig. 4)). Finally, the free text comments are listed. The students also have the opportunity, if necessary, to discuss the results in a personal discussion with the project leadership and to present their experiences in a reflection report (e-learning unit).

In the following, peer feedback was extended to students of the 1st semester in the model study course (321 out of 400 participated) in the winter semester 13/14 and additionally for students of the 3rd semester in the model course (216 out of 400 students participated). Since the summer semester 2016, it has also been open to students in the 5th, 9th and 10th semesters (180 students each). 

## 3. Results

### 3.1. Pilot phase 

#### General feedback

Amongst other things, inhibitions in expressing negative criticism were revealed. The reason given for this was uncertainty in the assessment, since no general rating scale was available for the individual categories [22]. While there was a desire for increased personal responsibility in the learning process, taking on responsibilities in everyday clinical routines, e.g. dealing with patients or giving feedback about students, was often considered too demanding:

“I was very angry about it, because it [referring to the gathering of feedback] again placed responsibility in our hands. We have to take care of it.” (Interview 1)

Often uncertainties in formulating constructive feedback face to face and teaching staff being short of time were also often mentioned by the students:

“So what came out in this feedback stuff: most of the people voicing criticism don’t want to make it personal. [...] There’s generally a tendency for most to say ‘we’re not trying to give you a dressing down.’ So they almost end up being overly positive.” (Interview 1)

I definitely benefited “from those who also commented on it. For example by saying, ‘that was quite good, I’’ll give you seven points for that. But you can improve on that if next time you try and...’ That’s constructive criticism which is useful. And not to say: ‘Yes, that was good, I’ll give you nine points for this.’ And leave it at that.” (Interview 5)

The perception of the feedback situation as an examination was deemed rather problematic by the students:

“It’s just [...] that [the feedback] creates a rather tense situation for the students. Because it’s obviously perceived as an examination and grading process.” (Interview 1)

“For the student, this does have something of an exam situation about it.” (Interview 2)

#### 3.2. Peer Feedback

##### General feedback

The implementation of anonymous peer feedback was initially also viewed with skepticism and restraint by the students. The uncertainties of the PY students already described (including inhibitions to express negative criticism, uncertainties about evaluation standards, uncertainty about learned feedback rules) were also mentioned in guided focus group interviews on the use of peer feedback from the students. Amongst other things the participants also tied the uncertainties in dealing with peer feedback to a hitherto non-existent trust culture at the location. In their evaluation on the feedback (summer semester 2013), individual students expressed a fear of hidden evaluations or spying by the Office of the Dean of Studies: 

“I felt that some were taking this opportunity to voice opinions they wouldn’t normally express. I find it unprofessional to include other people’s private matters when participating in a classroom assessment and then to disclose that to the Deanery. To be on the safe side, you should only enter what you actually experienced from someone but surprisingly you end up reading things you can’t believe were actually mentioned!“ (Feedback 12)

##### Free text comments

The free-text comments which were available in the peer feedback initially proved to be of little constructive use and partly served as an outlet for giving less constructive and sometimes also hurtful feedback under the protection of anonymity. This led to further reluctance by students and reduced the number of participants, whereupon in consultation with the student representatives future comments were reviewed prior to dissemination. As a result, free-text comments were reviewed by the project leaders and, if necessary, revised or deleted by the project coordinators. Here is an example of such a revision:

Before: “Also, your way of examining patients is really borderline [...] when faced with real patients you’re rather awkward, which isn’t that bad at the current level of competence. But it’s not ok that you try and cover that by acting tough and cool towards you fellow students. If you stop obsessing with pushing your ego and just focus on the things that are being asked there and then, all that criticism will drop away because you actually have all the skills to become a decent doctor.”

Afterwards: “Sometimes I have the impression that you are rather awkward when examining real patients, which isn’t that bad at our current level of competence. Nevertheless, I don’t think it’s necessary to cover this up by acting tough and cool when dealing with your fellow students. I think you have all the skills to become a decent doctor, so why don’t you try to accept it if you make a mistake in the future and focus on the things that are being asked there and then.” (Feedback 18)

Examples of student feedback removed by the project coordinators: “You stink!”, “You’re incompetent!”, “You’re such a dashing fellow!”

##### Interviews

As a result of the semi-standardized interviews with participants and non-participants of the first rounds of peer feedback, new approaches were developed to better inform and anchor the project, which should ultimately contribute to improved trust building:

- Linking the project with actual, visible people
- Personal project presentation by the project team in teaching events
- Creating a project homepage
- Creating an informative clip to explain the process of peer feedback
- Simplification and clearer organization of the peer feedback questionnaires
- Introduction of cooperative design
- Integration of the topic of “Feedback” at the beginning of the medical degree course

**Figure 5:**
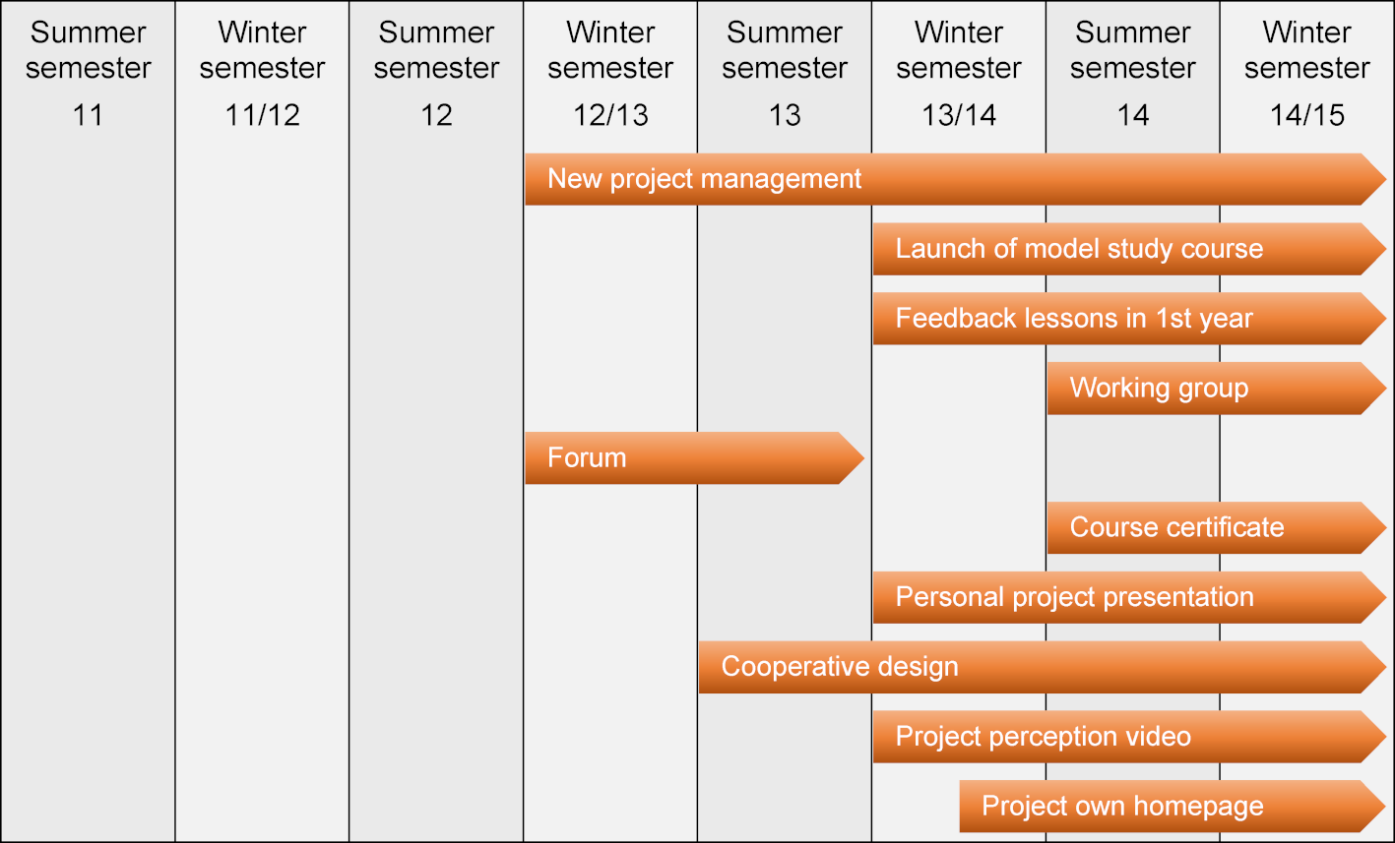
Date of introduction of project improvement measures

When which measures were implemented is shown in figure 5 (Fig. 5).

#### 3.3. Further development measures

The objectives of the teaching mission statement and the over-arching learning objectives for graduates of Düsseldorf University go beyond imparting medical knowledge and practical skills and developing the competences of students in all areas of medical practice and their personal development. On this basis, the students were given opportunities to gain accreditation for their commitments to certain competence areas, counting towards a faculty-internal “Medical Competencies” course certificate. Thus, for example, participation in the university mentoring program or the supervision of foreign students as well as commitment in external aid organizations are recognized as activities. The peer feedback was also included as an option counting towards the “Medical Competencies” course certificate in the 2014 summer semester. Students can acquire this certificate progressively between the 1^st^ and the 10^th^ semester.

**Figure 6:**
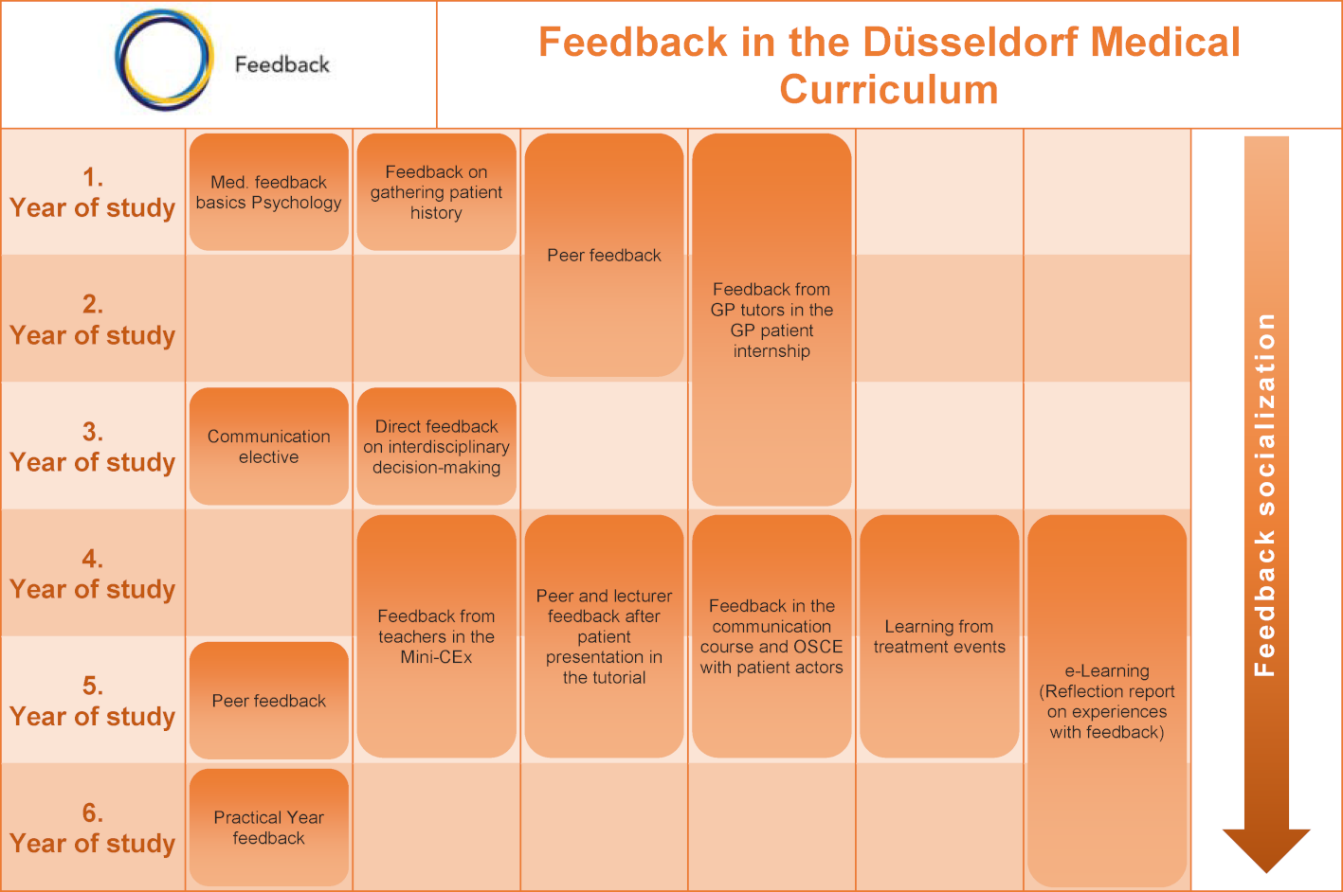
Courses in the Düsseldorf Medical Curriculum in which feedback is used

The steps towards implementing a feedback culture and responses from the peer feedback groups also showed that developing a culture of feedback first requires building a network of in-house networks to continually monitor feedback knowledge and methods against a dynamic reality to exchange new ideas. Based on these experiences, the project group aimed to integrate feedback even more strongly into the curriculum. The project group carried out a curricular mapping of the Düsseldorf Medical Curriculum on “Feedback” in order to determine the best options for anchoring a longitudinally coordinated feedback curriculum. An overview of the offers developed to date is shown in figure 6 (Fig. 6).

##### 3.3.1. Effectiveness of the further development measures

Inclusion of peer feedback in the curriculum: Acceptance evaluation

Peer feedback was regularly evaluated by the students. Based on the feedback from the questionnaire-based interviews, the concept was modified by implementing all suggestions for improvement proposed by the students (see 3.2). The following semester saw a change in the perception and acceptance of peer feedback, changes in quality (e.g. constructiveness of feedback) and rate of participation of offers (see table 1 (Tab. 1)). 

##### Improving the quality and activity of peer feedback

**Figure 7:**
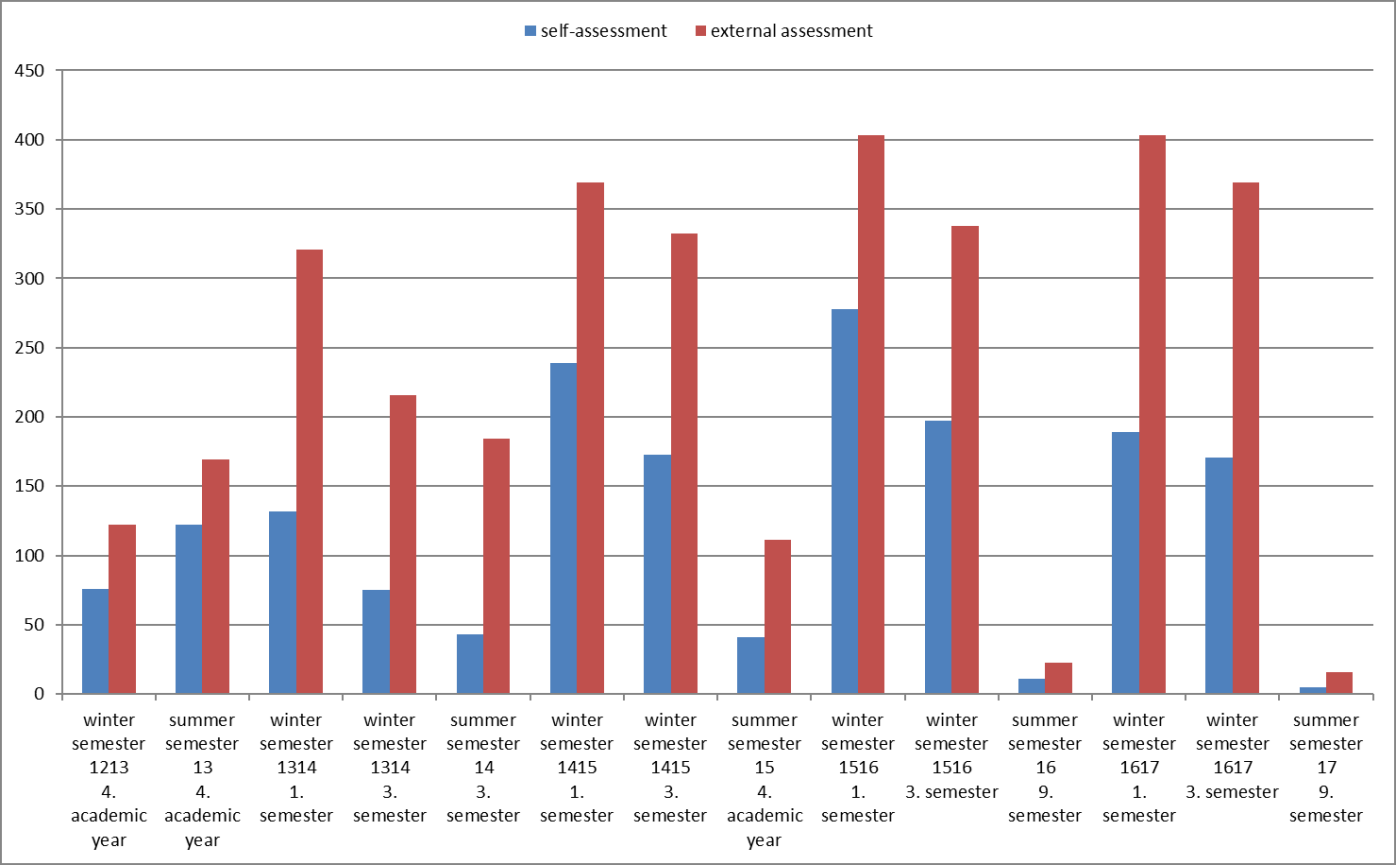
Development of the number of participants based on the example of self-assessments and external assessments in peer feedback over the years (maximum number of possible participants in the summer semester 180 students, 800 students in the winter semester).

**Figure 8:**
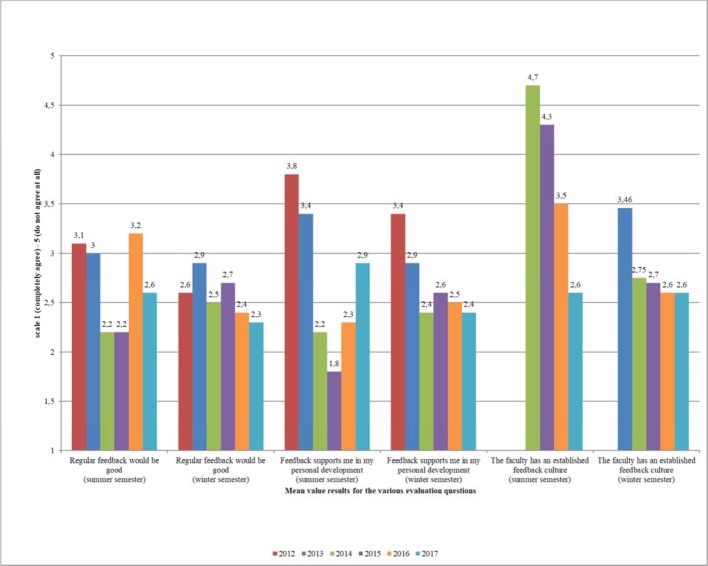
Student evaluation of peer feedback from 2012 to today on a five-level Likert scale (“completely agree” (1) to “do not agree at all” (5))

**Figure 9:**
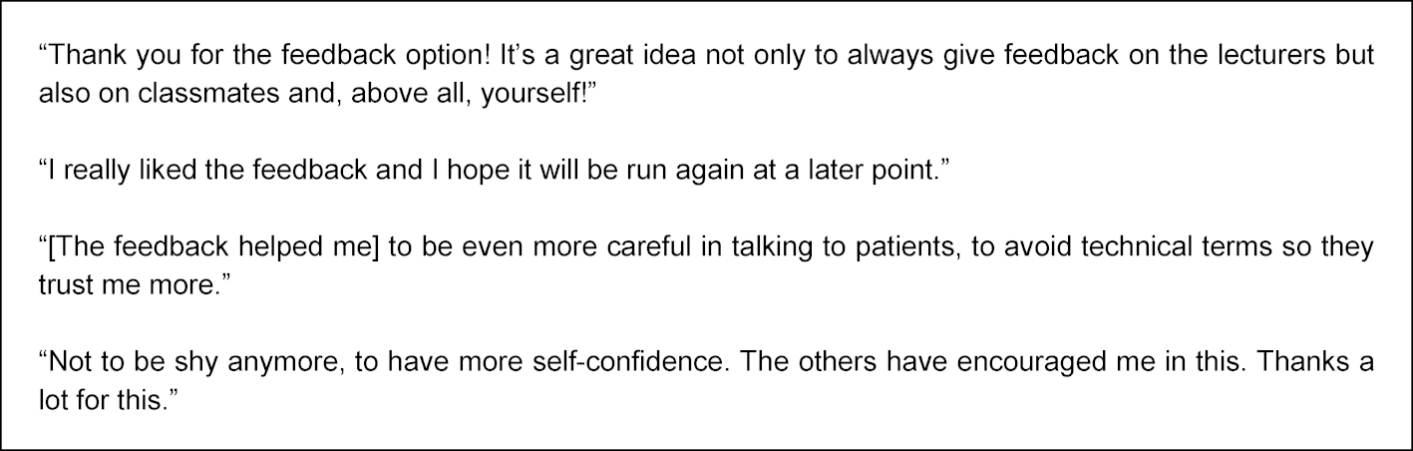
Written feedback (free text) of the students in the evaluation for peer feedback

Analysis of the free text comments submitted for their fellow students as part of peer feedback by two independent raters showed that the proportion of constructive free text comments in a cohort of the 2^nd^ year (beginning in winter semester 13/14) increased significantly by 11% to 99.4% compared to the previous year (t(3)=-3.79, p=0.04). The importance of feedback for personal development was rated in the project evaluation on a six-point scale (“completely agree” (1) to “do not agree at all” (6)) by students of this 2^nd^ year (n=109) scoring 2.53 (SD=0.97) on average; and 3.95 (SD=1.12) by the 4^th^ year (n=24) in the summer semester 2015. This difference was shown to be significant (t(405)=-3.78, p<0.05). On a five-point scale (I find the peer feedback “very useful” (1) to “not at all useful” (5)), in the winter semester 16/17 participants in peer feedback gave an average assessment of 1.9 (SD=1.0), in previous feedback (summer semester 2012) this had been 2.9 (SD=1.3). The significantly greater participation rate in peer feedback and the significantly more positive evaluation are shown schematically in figure 7 (Fig. 7) and figure 8 (Fig. 8). In addition, feedback from students provides information about any changes that may have been triggered (see figure 9 (Fig. 9)). Especially when assessing the feedback culture at the location, there is a steadily more positive assessment from semester to semester. 

##### Assessment of the culture of trust and feedback

Since the winter semester 14/15, a changed perception of the feedback culture is emerging between the various student cohorts. The culture of trust and feedback at the faculty was evaluated on a six-level Likert scale (“completely agree” (1) to “do not agree at all” (6)) by 109 students of the 2^nd^ year in the winter semester 15/16 with an average of 2.6 (SD=1.1), and 66 students of the 4^th^ year in the summer semester 2014 with 4.7 (SD=1.5) The difference was shown to be significant (t(4)=1.82, p=0.020). In addition, a comparison of all evaluations between the summer semester 2013 to the winter semester 17/18 showed a significant correlation between the academic year and participation in peer feedback (χ²=116.7, p<0.05). Students who have progressed further in their degree and who did not go through the feedback curriculum from the beginning perceived the feedback culture at the location as being worse. Statements from the student project evaluation in the 4^th^ year (66 students in the summer semester 2014) also showed that voluntary offers from the Deanery were viewed more critically in higher semesters [26].

## 4. Discussion and outlook

### 4.1. Discussion

The significant increase in participation in peer feedback and its significantly more positive evaluation suggest a more open attitude of the students towards feedback in the form offered. On the basis of the interview results a correlation with the above-described improvement measures for communication and presentation of the project and its change measures are likely. The mutually constructive and appreciative exchange between the participants and the project team may have contributed to increasing the acceptance of feedback and reducing emotional resistance [16]. It should be emphasized that students who were unable to complete the feedback curriculum from the outset for time reasons or who were unable to take part in each survey for personal reasons, perceived the feedback culture at the location to be worse as their studies progressed. Overall, however, in the model study course to date the attitude towards feedback has improved since the described measures were introduced. For students starting studies in the model study course (winter semester 2013/2014), an improvement of the attitude towards the feedback culture could amongst other things be linked to curriculum-based feedback sessions (such as role-playing with patient actors in medical psychology lessons) which took place as early as the first semester and which preceded the peer feedback. The introduction of peer feedback at the end of the semester, compared to previous cohorts (summer semester 12/13), this therefore offered students an opportunity to directly apply methods previously practiced in class. It is possible that feedback was seen as more natural and necessary in the context of medical studies from the outset and thus improved the dynamics at the faculty. Changing opinions of students regarding the desire for regular feedback and fluctuating number of participants (see figure 7 (Fig. 7) and figure 8 (Fig. 8)) can be explained by course overload. However, there were greater fluctuations among students in higher semesters who had not been integrated into a structured feedback curriculum from the first semester and who had not yet internalized the desirable culture of trust [13]. Of course, there is also the possibility that students in the first semester had a more positive attitude towards feedback from the outset, perhaps having had experience with feedback in school. This should be checked in future investigations. To what extent the quality improvement of student free-text comments towards constructive phrasing that was documented over the course of the project was a consequence of the project improvement measures cannot be substantiated reliably on the basis of the data. Filtering pejorative comments and the occasional cautious linguistic modification of the free-text comments, however, demonstrably went hand in hand with a decrease in complaints and criticism of the project as a whole. The option making peer feedback count towards a faculty-internal course certificate certainly contributed to an increase in the number of participants in terms of assessment-driven learning [27]. In the eyes of the students, having peer feedback count towards a course certificate in connection with assessment-driven learning seems to have a positive effect on the perception of the overall project. The resulting increased focus on the topic of feedback can only be helpful in reducing reluctance. This is also supported by the increasingly positive evaluation of peer feedback as a whole. 

Using the described concept, a structured implementation model [19] for a more holistic and socially embedded conceptualization of feedback was presented [20], which should be steadily expanded for the professional and personal support of students [8]. The presented measures can be regarded as a milestone on the way to a culture of feedback. 

#### 4.2. Recommendations - Lessons learned

Effective collaboration as well as personal commitment and perseverance of the project group proved to be key points in the implementation and development of feedback. When establishing new formats, modification requests and the interests of teachers and students should be elicited pro-actively and made central. The personal exchange between the project group and feedback users, i.e. students and teachers, turned out to be particularly useful. Obstacles were impersonal communication via email or measures implemented without consultation, which failed in everyday practice. Mentioning personal experiences in the area was especially helpful. It was also necessary to regularly study the feasibility of planned measures as well as to identify resistances (e.g. inhibitions in expressing negative criticism) and stumbling blocks (e.g. too time-consuming, too complex questionnaires) and their immediate elimination (e.g. offering training in dealing with feedback, shortening and simplifying the questionnaires). Strongly negative experiences of the students with feedback; refusal of the measures on the part of the faculty management as well as lecturers; and requiring too much time to elicit feedback in everyday university life can be regarded as natural implementation limits. 

Overall, there is a quality improvement of the feedback, which indicates the previously formulated project goals, such as the improvement of feedback competence [10] and thus the substantiation of feedback [12] as well as the improvement of social skills [9] have been met. Thus, feedback in the context of an established culture of trust with shared value creation [18] can be of great benefit in a supportive learning environment [17], [5], [14].

#### 4.3. Outlook

As the described intervention results show, the term feedback culture seems to be a sub-aspect of the culture of trust or of the learning organization. Increased transparency and linking the project with actual, visible people and opportunities for students to participate in decision-making also seem to increase acceptance and trust in the tools. The “Feedback” project group has formulated and initiated further steps in the development of a feedback curriculum:

##### 4.3.1. Implementation of direct feedback

Since the winter semester 15/16, direct feedback has been provided at the request of the students. A three-week study block in the 3rd year of the compulsory curriculum was chosen for this purpose in which the focus is on professional collaboration in an interdisciplinary team. Students work independently on realistic, complex patient cases portrayed by patient actors. Initially feedback was still restrained (for example, uncertainties in expressing criticism and in dealing with teamwork) strongly suggests that here too barriers to implementation must first be overcome and the concept further adapted to the needs of the users. It should be noted that because of its voluntary nature, peer feedback was not used by all students undergoing direct feedback. Furthermore, it should be examined whether students with participation in peer feedback have fewer difficulties in dealing with direct feedback and whether the experience with anonymous peer feedback, as described in the literature, had positive effects on the acceptance of feedback [2], [3], [4]. 

##### 4.3.2. Feedback training

Beneficial constructive feedback makes it necessary not only to deal with the topics but also to reflect on one’s own attitude towards feedback and trust in one’s own self-efficacy in dealing with feedback [28]. From this it can be deduced that ultimately the attitude of the feedback givers and recipients is decisive for the effectiveness of feedback [29]. Therefore the “Feedback” project group uses a newly developed training concept initially aimed at teaching staff, which not only conveys feedback rules but crucially also makes participants reflect on the way in which they handle feedback. Self-reflection in dealing with feedback is made familiar with the objective that this will have a qualitative effect on future feedback giving. 

## Notes

For the sake of readability, the simultaneous use of male and female forms is dispensed with. All references to people apply equally to both sexes.

## Competing interests

The authors declare that they have no competing interests. 

## Figures and Tables

**Table 1:**
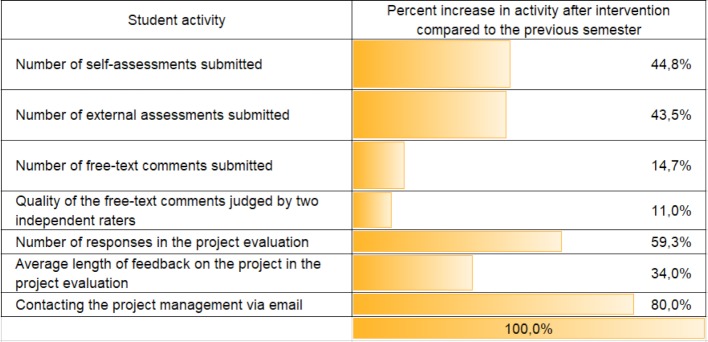
Percentage increase in student activities in the same cohort from winter semester 13/14 to winter semester 14/15 as part of the peer feedback after interventions by the project team in the summer semester 2014
